# Women’s knowledge and practices regarding urinary incontinence

**DOI:** 10.1186/s12889-025-25067-z

**Published:** 2025-11-06

**Authors:** Dina Adel Mostafa, Hanan I. Ahmed, Walaa A. Mohamed

**Affiliations:** Family and Community Health Nursing Department, Faculty of Nursing, Ain Shams University, Cairo, Egypt

**Keywords:** Knowledge, Practices, Urinary incontinence, Women

## Abstract

**Background:**

Urinary incontinence (UI) is a widespread condition affecting a large population globally. Women are disproportionately affected by UI, with the condition becoming more common with advancing age. UI is characterized as involuntary loss of urine during the bladder storage phase, which can significantly diminish a woman’s quality of life and impose substantial societal costs. Enhancing women’s knowledge is a key factor in reducing the incidence of UI.

**Aim:**

To assess the knowledge and practices of women concerning urinary incontinence.

**Methods:**

This descriptive analytical study was conducted at the urinary incontinence outpatient clinic at El-Demerdash Hospital in Cairo Governorate, Egypt. A purposive sample of 123 women was recruited for the study. Data were gathered using a structured interview questionnaire consisting of three parts: Part I, which focused on sociodemographic characteristics; Part II, which assessed women’s knowledge of urinary incontinence and pelvic floor muscle exercises; and Part III, which evaluated women’s self-reported practices regarding UI. Statistical analysis was performed using SPSS (Version 22.0). Descriptive statistics, including numbers, percentages, mean scores, and standard deviations, were used to present the data. The chi-square test was employed to examine the relationships between variables. At *p* < 0.01 there is a highly statistically significant difference between the variables.

**Results:**

The findings indicated that a majority of the women studied had a poor understanding of urinary incontinence (63.4%) and pelvic floor exercises (61.5%). Furthermore, a large proportion (71.5%) reported unsatisfactory practices related to urinary incontinence.

**Conclusion:**

The study established a highly significant relationship between women’s self-reported practices and their knowledge levels concerning urinary incontinence and pelvic floor muscle exercises. The study recommended implementing educational initiatives to increase women’s awareness of the importance of pelvic health and urinary incontinence.

**Supplementary Information:**

The online version contains supplementary material available at 10.1186/s12889-025-25067-z.

## Background

 According to the standard definition set out by the International Continence Society (ICS) and the International Urogynecology Association (IUGA), UI is described as a complaint of involuntary loss of urine experienced during the bladder storage phase [1]. Urinary incontinence (UI) is a medical condition that, despite its high prevalence, is frequently underreported and underdiagnosed due to associated social stigma and the misconception that it is a normal consequence of the aging process [2]. Obesity, numerous births (particularly vaginal delivery), a family history of incontinence, menopause, urinary tract infections, recurrent constipation, multiple sclerosis, diabetic neuropathy, and the use of pharmacological agents such as diuretics, antihypertensive medications, and anxiolytics are the predominant risk factors for urinary incontinence in women. These factors also increase the likelihood of developing incontinence as one ages [3].

Stress incontinence, urgency incontinence, mixed incontinence, overactive bladder and overflow incontinence, nocturnal enuresis, coital incontinence, and insensible incontinence are among the various types of urine incontinence that are known to exist [4]. This condition can profoundly affect a woman’s social functioning and emotional well-being. Research has demonstrated that women who suffer from UI are more susceptible to social isolation, diminished self-esteem, and heightened levels of anxiety. A woman’s whole quality of life is greatly impacted by her sexual health, which is inextricably tied to her self-esteem, emotional stability, and cognitive abilities. This condition plays a notable role in altering women’s sexual function behaviors, even when not directly related to sexual activity [2].

Pelvic floor muscle training (PFMT), an exercise program intended to reduce the symptoms of urine incontinence by exercising the pelvic floor muscles, is one of the main conservative treatment methods [5]. Exercises for strengthening the pelvic floor muscles involve tensing, holding, and then relaxing the muscles. The duration and intensity of pelvic floor muscular contractions are thought to be adequate to lessen undetected detrusor contractions [6].

The management of urine incontinence should rather employ a multidisciplinary strategy instead of depending on a singular profession. Community health nurses serve a critical front-line function in symptom assessment and educational delivery; nevertheless, optimal outcomes necessitate inputs from many disciplines [5]. Urogynecologists and urologists are essential for definitive diagnosis, specialized evaluations like urodynamics, and the provision of medical or surgical treatments. Furthermore, behavioral therapy and PFMT programs are typically overseen by physical therapists or physiotherapists and are routinely recommended by obstetricians, gynecologists, urologists, and nurse practitioner continence specialists, in addition to general healthcare providers. When required, pharmacists ensure the safe use of pharmaceuticals, dietitians provide guidance on weight management and nutrition, and psychologists can offer support for coping mechanisms and the impact on overall quality of life [7].

### Significance of the study

One significant issue in global health is urinary incontinence. Though the incidence rate among women aged 15 to 64 ranges from 10% to 30%, only a quarter of all women with this issue seek treatment, and the prevalence of UI rises with age. Between 20% and 40% of older women are thought to experience unintentional urine loss. Additionally, the World Health Organization (WHO) stated that approximately 200 million people worldwide suffer from urinary incontinence. More research should be done to determine the precise prevalence of UI in Egypt. Since the majority of Egyptian women are ashamed and refrain from seeking UI assistance, most do not report incontinence when they visit their healthcare providers, and most believe that UI is a normal part of aging and childbirth [8].

The prevalence rate of UI in particular Egyptian districts has been the subject of a few research studies. A study conducted in Assiut stated that 5.7%, 5.1%, and 11.4% of people had stress urinary incontinence, urge urinary incontinence, and mixed urinary incontinence, respectively, whereas 22.2% of the population had UI overall. Additionally, it was revealed that the overall prevalence of urine incontinence in Egypt was 54.8%, with stress urinary incontinence (SUI) accounting for 14.8% of cases [8].

Relevant studies’ findings state that urinary incontinence itself is not associated with mortality, and several factors indicating disability and frailty are likely to explain the association with mortality. It substantially affects morbidity and severely reduces the quality of life for affected women. Urinary incontinence and its associated risk factors lead to a cascade of adverse consequences that negatively impacts survival. These results highlight how crucial it is to screen for mortality in relation to urinary incontinence in clinical practice, which is why the researcher should evaluate women’s awareness and actions about urine incontinence [9].

## Methods

### Study Design

 A descriptive analytical study was employed to assess women’s knowledge and practices regarding urinary incontinence. The research was conducted at the outpatient clinic specializing in urinary incontinence at El-Demerdash Hospital, Ain Shams University, Cairo, Egypt. A purposive sample of 123 women was recruited for the study.

### The inclusion criteria

Were all women aged 18 years or older who were diagnosed with any type of urinary incontinence (stress, urge, or mixed) by a physician.

### The exclusion criteria

 Women with immunity diseases such as multiple sclerosis and myasthenia gravis were excluded, as such conditions could potentially confound the typical clinical presentation of UI.

Using the Epi Info 7 program for sample size calculation, setting the confidence level at 95%, the margin of error at 5.5%, and the total population size at 180, a sample size of 123 women was sufficient to detect an expected prevalence rate of good knowledge of 50% [10].

## Research questions

- Q1. What is the level of women’s knowledge regarding urinary incontinence?
- Q2. What is the level of women’s knowledge regarding pelvic floor muscle exercise?
- Q3. What is the level of women’s self-reported practices related to urinary incontinence?
- Q4. Is there a relationship between women’s knowledge and their self-reported practices regarding urinary incontinence?

### Instruments: a structured interview questionnaire was utilized for data collection, consisting of three distinct parts

- *Part I* was designed to collect sociodemographic data, including age, profession, marital status, education level, income, and residence.
- *Part II*, which assessed women’s knowledge regarding urinary incontinence and pelvic floor muscle exercises, was developed by the researchers based on a comprehensive review of the literature [11–13]. These studies serve as a theoretical and cultural basis for item development. This instrument was created in Arabic and comprised 14 multiple-choice items across two sections, covering topics such as the meaning, types, risk factors, symptoms, complications, and treatment of UI, as well as the definition, types, application, and importance of pelvic floor exercises. Minor wording modifications were made to reflect the local Egyptian cultural context without altering the core meaning of the items. An appendix (Additional File 2) contains the original and revised English versions. The instrument also included questions regarding the source of information.
  The scoring system assigned 2 points for a complete response, 1 point for an incomplete response, and 0 points for a wrong or unknown response. The total score was 50 points, with the following categories for knowledge levels: poor knowledge (≤ 25%), fair knowledge (50%–75%, or grades 26–37), and good knowledge (≥ 75%, or grades 38–50) [14]. The percentage cutoffs were established a priori based on similar studies.
  Its psychometric properties were rigorously assessed, demonstrating high internal consistency reliability with a Cronbach’s alpha of 0.853. The scale also showed strong test-retest reliability with a correlation of 0.88. The exploratory factor analysis further reinforced the scale’s validity, with initial factor loadings ranging from 0.50 to 0.72 before rotation and improving to between 0.55 and 0.87 after varimax rotation, all surpassing the 0.35 threshold. Additionally, the Kaiser-Meyer-Olkin measure of sampling adequacy was 0.88, demonstrating that the data were highly suitable for factor analysis.
- *Part III *evaluated women's self-reported practices related to urinary incontinence. This instrument was adapted from Porto et al. [15] and consisted of 10 yes/no questions. The adaptation process involved rewording some items, 22 and 28, to suit the local Egyptian cultural context without altering the core meaning. An appendix (Additional File 3) contains the original and revised English versions. The scoring system comprised ten items with values ranging from Yes=1 to No=0 for positive items and vice versa, Yes=0 to No=1 for negative ones (“limit your daily fluid consumption” and “avoid sexual contact”). Each item's score was added up and transformed into a percentage score in the manner described as follows: Satisfactory reported practice ≥ 75% and unsatisfactory reported practice ≤ 75% [16]. The adapted instrument demonstrated high internal consistency reliability with a Cronbach’s alpha of 0.97. It also showed robust test-retest reliability with a correlation coefficient of 0.85, and exploratory factor analysis further reinforced the scale’s validity, with initial factor loadings ranging from 0.55 to 0.75 before rotation, improving to between 0.60 and 0.90 after varimax rotation, all surpassing the 0.40 threshold. These factors accounted for a cumulative variance of 72.50%. Additionally, the Kaiser-Meyer-Olkin measure of sampling adequacy was 0.90, demonstrating that the data were highly suitable for factor analysis.

### Data collection phases

The study’s protocol received formal approval from the nursing faculty at Ain Shams University. A pilot study was conducted with 12 participants (10% of the sample) to identify any issues and make minor revisions for clarity and flow. The pilot participants were excluded from the final analysis of the main study to prevent potential bias, in accordance with best practice. Data collection took place over a three-month period from January 15 to April 15, 2025. The questionnaire’s face and content validity were evaluated by a jury of three specialists from the family and community health nursing staff at Ain Shams University. Written informed consent was obtained from each participant after the study’s purpose was explained. Each interview took 20 to 30 min, and for illiterate women, the questions were read aloud by the investigator, who then documented the responses.

### Statistical analysis

The collected data were initially coded, entered, and verified using the Statistical Package for the Social Sciences (SPSS), Version 22.0. Descriptive statistics, including frequencies, percentages, mean scores, and standard deviations, were utilized to present the data. To investigate the relationships between variables, the chi-square test was employed. A p-value of less than 0.01 was established as the threshold to indicate a highly statistically significant difference in the analysis.

## Results

Table 1 indicates that 46.3% of the women in the study were between the ages of 41 and 50, with a mean age of 53.316 ± 12.713; 78.9% of them were workers, 70.7% of them were married, 69.9% of them had a secondary educational level, 61% of them had an insufficient income level, and 74% of them were from urban areas.

**Table 1:** The study’s women’s frequency distribution regarding sociodemographic traits (*n* = 123)

Personal data	No	%
Age		
less than 40 years	38	30.9
41–50 years	57	**46.3**
51 years and more	28	22.8
Mean ± SD	**53.316 ± 12.713**	
Profession		
Worker	97	**78.9**
Housewife	26	21.1
Marital status:		
Married	87	**70.7**
Divorced	26	21.1
Widow	8	6.5
Unmarried	2	1.6
Level of education		
Primary	6	4.9
Middle school	4	3.3
Secondary	86	**69.9**
University	22	17.9
Post-university	5	4.1
Income level		
Sufficient	48	39.0
Insufficient	75	**61.0**
Residence		
Rural	32	26.0
Urban	91	**74.0**

**Fig. 1:**
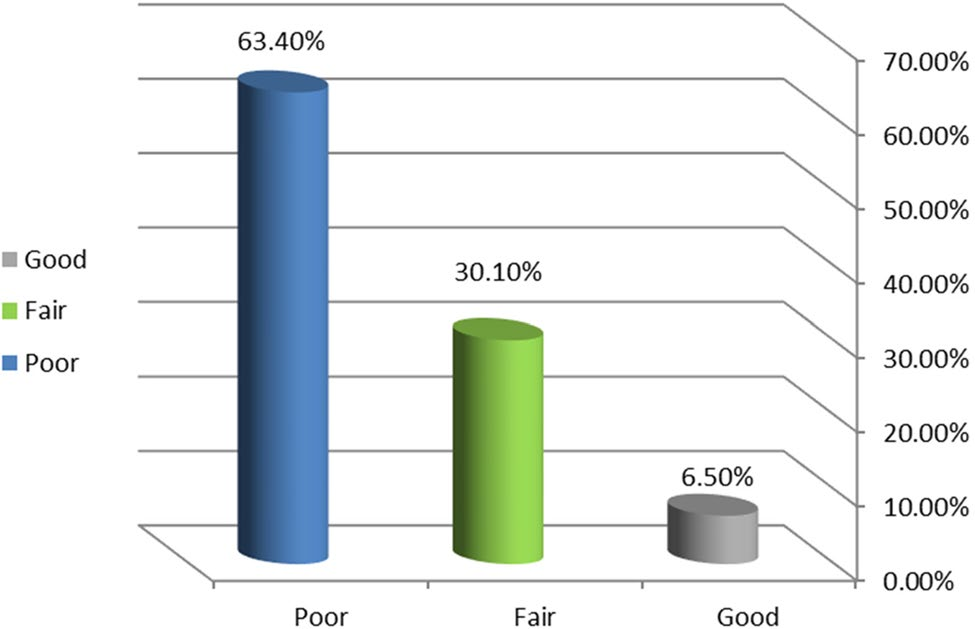
Distribution of the women in the study's total urinary incontinence knowledge. (n=123)

Figure 1 illustrates that 63.4% of the women had a poor knowledge level, while 30.1% had fair knowledge and only 6.5% had good knowledge.

**Fig. 2:**
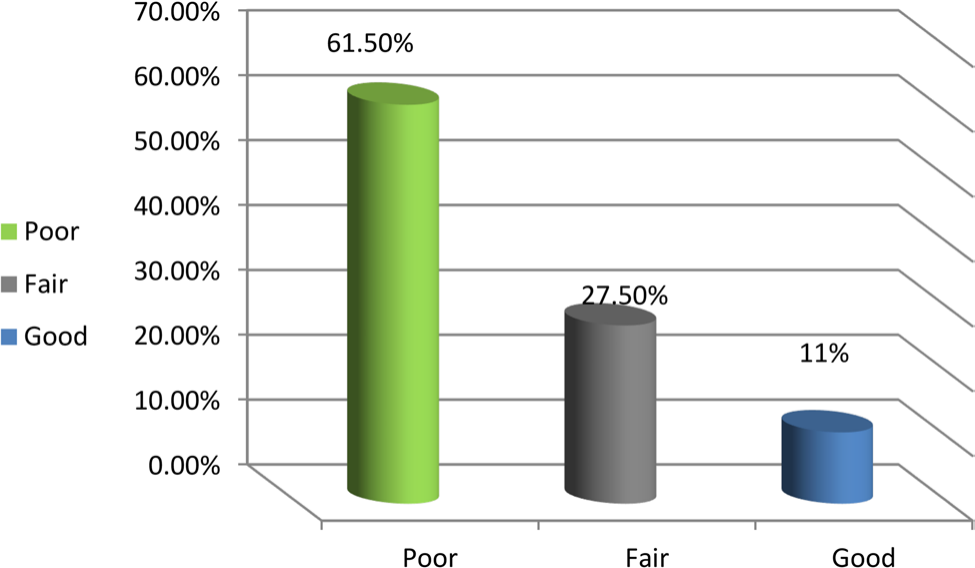
Distribution of the women in the study's total pelvic floor exercise knowledge (n=123)

Figure 2 shows that 61.5% of the women had poor knowledge of pelvic floor exercises, with 27.5% having fair knowledge and 11% having good knowledge.

**Fig. 3:**
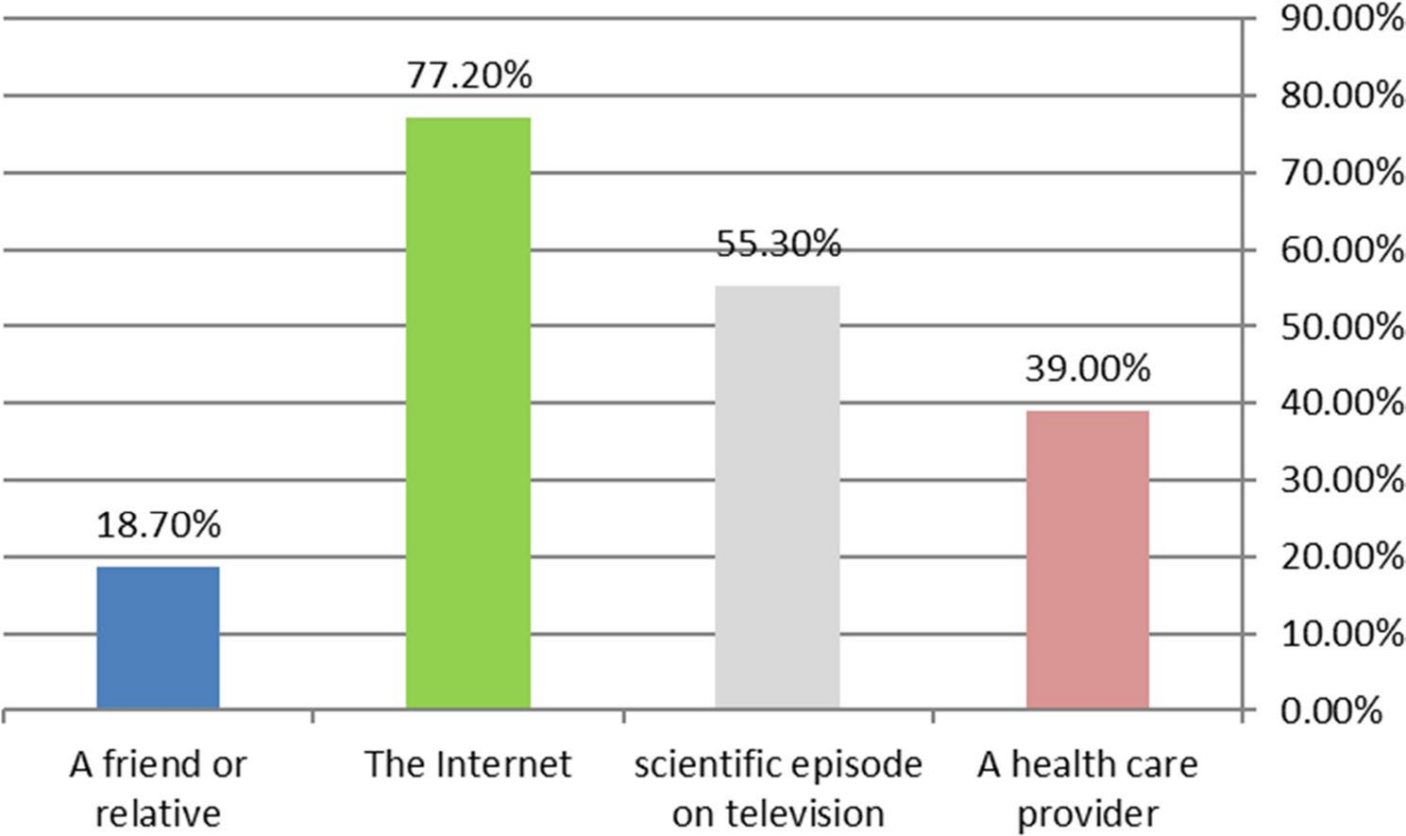
Information source regarding urine incontinence and pelvic floor exercises

 Figure 3 reveals that 77.2% of the women in the study primarily had their information from the internet. Other sources included scientific television episodes (55.3%), healthcare providers (39%), and friends or relatives (18.7%).

 Table 2 finds that 89.4% of the women in the study "change underwear several times during the day" and "change sanitary pads several times during the day." Also, 82.9% of them "use absorbent materials and hygiene products" and "use dark and loose clothing," while 82.9% of them agree about "practicing pelvic floor exercises regularly."

**Table 2:** The frequency distribution of the general practices reported by the women under study (*n* = 123)

Items	Yes		No	
	No	%	No	%
Go to the bathroom many times during the day	98	79.7	25	20.3
Avoid activities that require physical effort	90	73.2	33	26.8
Use absorbent materials and hygiene products	102	**82.9**	21	17.1
Use dark and loose clothing	102	82.9	21	17.1
Change underwear several times during the day	110	**89.4**	13	10.6
Reduce fluid intake during the day	93	75.6	30	24.4
Change sanitary pads several times during the day	110	89.4	13	10.6
Follow a balanced diet to reduce weight	101	82.1	22	17.9
Avoid sexual intercourse	75	61.0	48	39.0
Practice pelvic floor exercises regularly	21	17.1	102	**82.9**

**Fig. 4:**
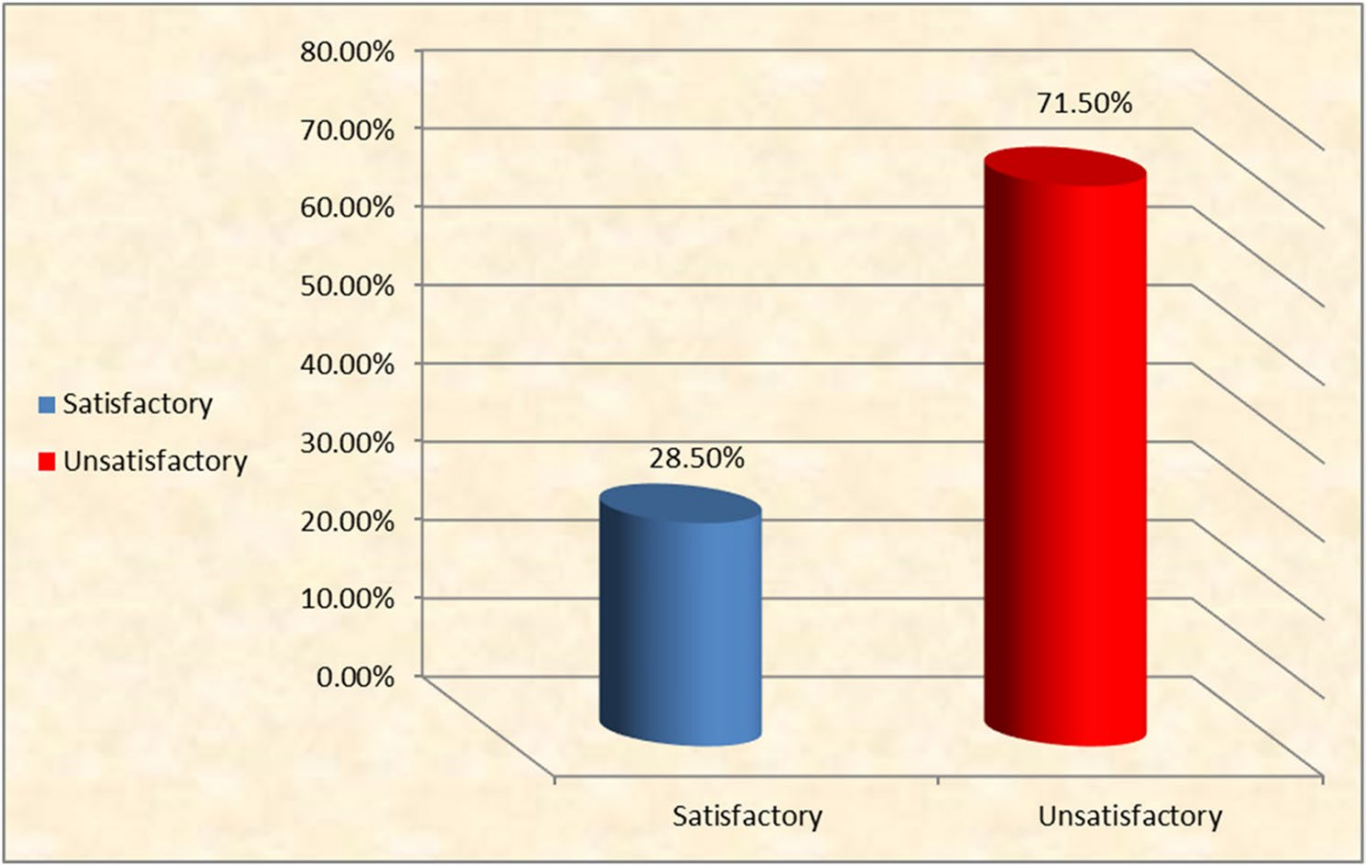
Distribution of the studied women’s total reported practices related to urine incontinence (n =123)

Figure 4 displays the total distribution of stated practice. It was found that 71.5% of the women had an unsatisfactory practice level, while 28.5% had a satisfactory level.

Table 3 demonstrates a statistically significant relationship between the women’s self-reported practices and their level of knowledge about pelvic floor muscle exercise and urine incontinence (X² = 22.11, *P* < 0.001).

**Table 3:** Relation between studied women's knowledge level and reported practices level regarding urine incontinence and pelvic floor exercises (n=123)

Variables		Total practices				X^2^	*P*
		Satisfactory		Unsatisfactory			
		No	%	No	%		
Total knowledge	Good	6	4.9	2	1.6	22.11	< 0.001
	Fair	37	30.1	0	0.0		
	Poor	45	36.6	33	26.8		

## Discussion

The findings of this study confirm that urinary incontinence is a highly prevalent condition that significantly affects the physical and social activities of women, in addition to their interpersonal interactions. Enhancing women’s knowledge regarding UI is a matter of considerable clinical and social significance, as access to evidence-based knowledge, pharmacological interventions, and physiotherapy can markedly improve symptoms and disease severity Hammad [17]. Women may withdraw socially due to fears of incontinence, urine odor, and soiling their clothing, resulting in social isolation and impediments to physical activity stemming from embarrassment and psychological discomfort. Developing culturally sensitive and healthy educational interventions is essential to enhance clinical outcomes and social participation [18].

The sociodemographic profile of the study participants, with a mean age of 53.3 ± 12.7 years, aligns with findings from other studies conducted in both national and international contexts. These results were supported by a study conducted by Yağmur & Gül [19] in Batman City, which revealed that the women’s mean age was 51.6 (8.8) (40–69 years old). Also, these findings were also confirmed by a study by Elmorsey et al. [20] in Egypt, which found that 43.6% of the women in the study were between the ages of 31 and 40, with a mean age of 34.9 ± 8.2.

The age distribution in these studies demonstrates that perimenopausal and postmenopausal stages are critical for developing UI. Also, this may be explained by the estrogen levels starting to decline in this age group, which affects the urethra, bladder, and pelvic floor muscles, making the tissues thinner, weaker, and less elastic.

Regarding occupation, the finding that over three-quarters of the participants were workers is consistent with a study in Turkey by Erenel & Ozdemir [21] (51.5% were employees) but contrasts with research in Malaysia by Fauzey et al. [22], where most women were housewives (52.6%).

From the investigator’s point of view, this may be because urinary incontinence is more common and can be exacerbated among working women due to occupational factors, such as strenuous physical activity, prolonged awkward positions, and limited access to toilets, which increase the risk of leaks, stress, and negative impacts on concentration and performance status. In addition, UI symptoms are more likely to cause a woman to seek professional assistance.

The large proportion of married women (70.7%) in the sample aligns with results from Abu Raddaha & Nasr [23] in Port Said City, Egypt, and revealed that 93.5% of women were married. Also, this result was similar to the study performed by Elattar et al. [24] conducted at Komhamada General Hospital, Egypt, whose study revealed that 75.7% of them were married.

From the investigator’s point of view, this may be due to the prevalence of risk factors such as vaginal delivery and a history of gynecological or pelvic surgery in this population.

In terms of education, the present survey showed that almost two-thirds of them had completed secondary school. These findings were in line with research by Elmorsey et al. [20], which discovered that 44.5% of the women in the sample had completed secondary school. According to research by Abu Raddaha & Nasr [23], 49.7% of women had completed secondary school.

Concerning income, the recent study demonstrated that less than two-thirds of them had an insufficient income level. These results were supported by Elattar et al. [24], who demonstrated that 62.8% didn’t have enough income. Also, we’re congruent with Moustafa [16]; a study done in Dakahlia Governorate, Egypt, revealed that 53.1% of women had enough income. This may result from the participants’ education and income levels mirroring common cultural and economic circumstances. Whereas, female marriage in some Egyptian cultures is more valuable than holding a higher educational level.

Concerning place of residence, our study found that nearly three-quarters of them were from urban areas. These findings ran counter to Zhang et al. [25] in Taiyuan and revealed that 36.4% of women were from rural areas. In addition, this finding contradicted a study done in Minia, Egypt, published by Mohammed et al. [26], which found that 54% of women were from rural areas.

From the investigator’s perspective, this may be due to the higher impact of UI on the active urban lifestyle compared to a rural one. Urban life often involves more social engagement and employment, making the symptoms of UI more disruptive and thus more likely to prompt a woman to seek professional help.

The study revealed that under two-thirds of the women have poor information regarding incontinence, less than one-third exhibited fair knowledge, and a small minority demonstrated good knowledge. These results were consistent with research by Li et al. [27] in Shenzhen, China, which indicated that women lacked information about urinary incontinence. In contrast to the findings of Wieczerzycka & Skwiot [28] in Poland, these data showed that 43% of women said they knew enough about urinary incontinence. In addition, this result contradicted Ribeiro et al. [29], who found that most of the women (89.6%) had good knowledge regarding urinary incontinence.

From the investigator’s point of view, this may be due to women’s misperception of urinary incontinence as a normal aspect of aging or childbirth, leading to a lack of urgency in seeking information or treatment. This misconception can delay intervention and contribute to the persistence of symptoms.

According to the current study, a few of the women in the study had good knowledge levels about pelvic floor exercises, whereas less than two-thirds had poor information, and over 25% had fair knowledge. These results were in contrast with a survey done by Tennfjord et al. [30]. Performed in northwest Ethiopia, it revealed that 42.0% of pregnant women had good knowledge. Furthermore, this result contradicted a study in Malaysia done by Gorantla Mamatha & D’souza [31], who found that 49.6% of participants demonstrated good knowledge regarding pelvic floor muscle exercises.

From the investigator’s perspective, this may be attributed to low educational level and limited access to information, which can negatively impact knowledge about pelvic floor disorders and exercises.

Regarding the information source, more than three-quarters of the women in the study primarily had their information from the internet. These findings align with research by Elmahgoub et al. [32], in Jordan, which demonstrated that 38% of women stated that YouTube or other social media platforms were the source of their information. Additionally, it differs from research conducted in Barahachhetra by Basnet et al. [33], which found that 81% of women acquired their knowledge from friends.

From the investigator’s point of view, this may be due to the availability of internet in each home, besides faster and easier information access.

The current study showed that the majority of the studied women “change underwear several times during the day” and “change sanitary pads several times during the day.” Also, most of them “use absorbent materials and hygiene products” and “use dark and loose clothing,” while the majority of them agreed about “practicing pelvic floor exercises regularly.”

These findings were similar to those of the study performed in Egypt by Elsayed et al. [34], which revealed that 55.9% of women change underwear frequently and put on absorbable pads and underwear. Additionally, a study conducted in Abha, Saudi Arabia, by Othman et al. [35] revealed that 57% of women did not perform Kegel exercises, supporting similar findings.

From the investigators’ perspectives, this may be attributed to women seeking hygiene behavior and women desiring to prevent skin irritation and reduce the risk of urinary tract infections.

The current study revealed that more than one quarter of the studied women had a satisfactory practice level regarding urinary incontinence, whereas less than three quarters of them practiced this topic to an unsatisfactory degree. Similarly, three studies support these results [29, 34, 36]. Firstly, Ribeiro et al. [29] found that the women had inadequate practice both to prevent (89.2%) and to treat (78.8%) urinary incontinence. Secondly, Elsayed et al. [34] in Egypt concluded that 57.4% of women had unsatisfactory practice levels regarding urinary incontinence. Thirdly, da Silva Coelho et al. [36] demonstrated that only 29.5% adopted treatment practices, while preventive practices were carried out by only 16.4%.

From the investigators’ perspectives, this may be attributed to lower educational attainment and certain cultural backgrounds that are associated with poor knowledge and practices of urinary incontinence.

According to the current study, there was a statistically significant relation between the women’s stated practices and their level of knowledge about pelvic floor muscle exercise and urine incontinence.

This outcome aligned with the findings of da Silva Coelho et al. [36] in Brazil, which indicated a correlation between adequate knowledge and effective preventative and therapeutic activities, as well as between inadequate knowledge and inadequate practices. Additionally, this result agreed with Gomaa et al. [37], a study conducted in Tanta, Egypt, which revealed that there was a significant positive correlation between total knowledge scores and total practice scores regarding urinary incontinence. From the investigators’ perspectives, this relationship is likely driven by the fact that women who comprehend the risks and consequences of UI are more motivated to engage in health-promoting behaviors.

### Strengths and weakness

This study addresses a sensitive and researched issue in the Egyptian context, employing a validated instrument that ensures the reliability and accuracy of collected data. However, the exclusion of women with immunity diseases, such as multiple sclerosis and myasthenia gravis, limits the generalizability of the findings to all women with urine incontinence. This exclusion may have resulted in selection bias. Because of this, it’s possible that the prevalence and severity of urine incontinence were underestimated, and comparisons with other studies that involve this population should be conducted with caution.

### Limitations

The study was performed at a singular outpatient clinic, hence constraining the generalizability of the results. Future research with a larger sample size across multiple sessions to improve the generalizability of results.

## Conclusion

The current study indicated that under two-thirds of the women had poor knowledge regarding urinary incontinence and pelvic floor exercises. This deficiency in knowledge was reflected by unsatisfactory practice levels regarding urinary incontinence management in more than two-thirds of the participants. Furthermore, a highly statistically significant relationship was established between the women’s self-reported practice and their level of knowledge regarding urine incontinence and pelvic floor muscle exercises.

## Supplementary Information

Supplementary Material 1

Supplementary Material 2

Supplementary Material 3

## Data Availability

Data will be available upon reasonable request.

## Abbreviations

UI: Urinary incontinence
PFMT: Pelvic floor muscle training
WHO: World health organization
SUI: Stress urinary incontinence
