# Supplementary material for: Women’s knowledge and practices regarding urinary incontinence
Source: BMC Public Health. 2025 Nov 6;25:3812. doi: 10.1186/s12889-025-25067-z (PMC12590743; doi:10.1186/s12889-025-25067-z)
Supplement: Supplementary file 1 — Supplementary Material 1 [file 12889_2025_25067_MOESM1_ESM.docx]

**Women’s knowledge and Practices Regarding**

**Urinary incontinence**

^Note: All data is confidential and will only be used for scientific research.^

P**art I:** Sociodemographic Data

**Age :-1**

(**Profession:** A. Worker ( ) B. Housewife( **-2**

**Marital status:** A. Married ( ) B. Divorced ( ) C. Widow ( ) D. Unmarried**-3**

**4-Level of education:** A. Primary ( ) B. Middle school ( ) C. Secondary ( ) D. University ( ) E. Post-university ( )

( ) **Income level:** A - Sufficient ( ) B - Insufficient **–5**

**6-Residence:** A – Rural ( ) B – Urban ( )

**Part II:** Women’s knowledge regarding urinary incontinence and pelvic floor muscle exercises.

**section (I),** Women’s knowledge about incontinence in the urine.

**7-**Is the abrupt, uncontrollable flow of pee known as urinary incontinence?

A - Yes ( ) B - No ( ) C - I don’t know.

8- Which kinds of urine incontinence are you familiar with?

A. Exhausting (with work). B. Urgency (urine leaks right after the need to urinate) C. Both combined. D-Everything mentioned above E. None of the aforementioned F. I don’t know.

9-What are the reasons behind incontinence in the urine?

A. Weakness in the muscles of the bladder. B. The pelvic muscles are weak. C. Both of them are weak. D. None of the aforementioned. E. I don’t know.

10-What are the contributing reasons to incontinence?

A. Chronic illnesses B. Psychological tensions and emotions C. Recurrent pregnancy D. Old age E. Obesity F. Everything mentioned above. G: None of the aforementioned H. I don’t know**.**

11-Which symptoms are connected to incontinence of the urine?

A. Incapacity to regulate urination. B. The inability to contain pee over extended periods of time C. Repeated urination with a tiny quantity of urine, nearly in drops D. Urine drops that leak right after urination. E. I don't know.

12-What consequences may urinary incontinence cause?

A. Problems that are physical. B. social. C. Complications of the mind. D. Economic complications. E. Psychological complications. F. I don’t know.

13-How can urinary incontinence be avoided?

A. Preserving the urinary system's integrity. B. Preventing obesity. C. Taking care of the vaginal dilatation site during childbirth. D. Time off in between pregnancies. E. Other Remember.

14-Which techniques are used to cure incontinence?

A. A prescription for medication written under a doctor's supervision. B. The surgical procedure. C. Constant pelvic floor exercises. D. I don’t know.

**(section II),** knowledge of women about exercising the pelvic floor muscles.

15-Does the capacity to control pee voluntarily result from pelvic floor exercises that strengthen the muscles of the bladder and pelvic floor by drawing them inward for a while?

 A - Yes ( ) B - No ( ) C - I don’t know.

16-What significance do these activities have?

A. One strategy to prevent urine incontinence is to strengthen the muscles in the pelvis and bladder. C. One method of treating incontinence in the urine D. Other Remember.

17-What kinds of exercises target the pelvic floor?

A. Kegel exercises to tighten and release the pelvic muscles B. Squatting C. The location of the table or its evaluation D. Position of the bird E. I don’t know.

18-Which times are ideal for using it?

A. To strengthen the vaginal muscles after giving birth B. To treat urinary incontinence during pregnancy. C. Following vaginal dilatation or patching procedures. D. I don’t know.

19-What are the conditions that exclude performing these exercises?

A. Since exercise has no negative side effects, there are no contraindications. B. The existence of serious infections or sores behind the bladder or vagina. C. Experiencing discomfort while exercising. D. I don’t know.

20-When is it possible to practice these exercises?

A. Since exercise has no negative side effects, there are no contraindications. B. The existence of serious infections or sores behind the bladder or vagina. C. Experiencing discomfort while exercising. D. I don’t know.

21-What is the source of your information about urine incontinence and pelvic floor exercises?

A. A health care provider. B. A scientific episode on television. C. The Internet. D. A friend or relative.

**Part III:** Women’s stated practices about incontinence.

| practices | Yes | No |
| --- | --- | --- |
| 22-go to the bathroom many times during the day. |  |  |
| 23-Avoid activities that require physical effort. |  |  |
| 24-Use absorbent materials and hygiene products. |  |  |
| 25-Use dark and loose clothing. |  |  |
| 26-Change underwear Change underwear. |  |  |
| 27-Limit your daily fluid consumption. |  |  |
| 28-Alternate sanitary pads multiple times during the day. |  |  |
| 29-Eat a well-balanced diet to lose weight. |  |  |
| 30-Avoid sexual intercourse. |  |  |
| 31-Perform pelvic floor exercises on a regular basis. |  |  |
