# Supplementary material for: Women’s knowledge and practices regarding urinary incontinence
Source: BMC Public Health. 2025 Nov 6;25:3812. doi: 10.1186/s12889-025-25067-z (PMC12590743; doi:10.1186/s12889-025-25067-z)
Supplement: Supplementary file 2 — Supplementary Material 2 [file 12889_2025_25067_MOESM2_ESM.docx]

**Appendix 1.**

Items underwent a minor wording change to reflect the cultural and linguistic context of Egyptian women.

**Item 9 underwent a minor wording change""**

**Original** **English version:**

Can involuntary loss of urine be caused by several easily treatable medical conditions?

**Revised English version**:

What are the reasons behind incontinence in the urine?

**Item 12 underwent a minor wording change""**

**Original English version:**

Do most people who currently have involuntary urine loss live normal lives?

**Revised English version**:

What consequences may urinary incontinence cause?

**Item 16 underwent a minor wording change""**

**Original English version:**

What do you think pelvic floor muscle training is used for?

**Revised English version**:

What significance do these activities have?

**Item 21 underwent a minor wording change""**

**Original English version:**

Who would you go to, to learn about pelvic floor exercises?

**Revised English version**:

What is the source of your information about urine incontinence and pelvic floor exercises?
