# Supplementary material for: Women’s knowledge and practices regarding urinary incontinence
Source: BMC Public Health. 2025 Nov 6;25:3812. doi: 10.1186/s12889-025-25067-z (PMC12590743; doi:10.1186/s12889-025-25067-z)
Supplement: Supplementary file 3 — Supplementary Material 3 [file 12889_2025_25067_MOESM3_ESM.docx]

**Appendix 2. Minor Adaptation to Item 22 and 28**

During the adapting process, Items 22 and 28 underwent a minor wording change to reflect the cultural and linguistic context of Egyptian women.

**Item 22 underwent a minor wording change""**

**Original** **English version:**

Going to the toilet very often.

**Revised English version**:

Go to the bathroom many times during the day.

**Item 28 underwent a minor wording change""**

**Original English version:**

Wear pads to keep dry.

**Revised English version**:

Alternate sanitary pads multiple times during the day.
